# Association between cholesterol levels and dementia risk according to the presence of diabetes and statin use: a nationwide cohort study

**DOI:** 10.1038/s41598-022-24153-1

**Published:** 2022-11-12

**Authors:** You-Bin Lee, Min Young Kim, Kyungdo Han, Bongsung Kim, Jiyun Park, Gyuri Kim, Kyu Yeon Hur, Jae Hyeon Kim, Sang-Man Jin

**Affiliations:** 1 Division of Endocrinology and Metabolism, Department of Medicine, Samsung Medical Center, Sungkyunkwan University School of Medicine, 81 Irwon-Ro, Gangnam-Gu, Seoul, 06351 Republic of Korea; 2 Department of Statistics and Actuarial Science, Soongsil University, Seoul, Republic of Korea

**Keywords:** Dementia, Dyslipidaemias

## Abstract

We explored the association between cholesterol levels and dementia risk according to the presence of diabetes and statin use. In this population-based longitudinal cohort study, the Korean National Health Insurance Service datasets (2002–2017) were used. Among individuals aged ≥ 40 years who underwent health examinations in 2009 (N = 6,883,494), the hazard of dementia was evaluated according to cholesterol levels. During a median 8.33 years, 263,185 dementia cases were detected. In statin non-users with or without diabetes, the hazards of all-cause dementia were highest for those in the lowest quartile or quintile of low-density lipoprotein-cholesterol (LDL-C) level, showing an inverted J-shaped relationship. Among statin users with or without diabetes, an advance in LDL-C group was associated with an increase in hazards of all-cause dementia. In statin users with diabetes, even very low LDL-C level was not associated with an increased risk of all-cause dementia. Although there was a seemingly paradoxical association between low LDL-C level and dementia risk in statin non-users, the trend was not observed in statin users and is not likely to be clinically relevant. Rather, an advance in LDL-C levels was associated with an increase in the hazard of all-cause dementia in statin users, regardless of the presence of diabetes.

## Introduction

Based on overwhelming evidence that statins reduce the risk of atherosclerotic cardiovascular disease (ASCVD), guidelines on ASCVD risk management have endorsed the intensification of statin therapy based on the ASCVD risk^1^. Emerging evidence indicates that a more aggressive lowering of low-density lipoprotein-cholesterol (LDL-C) and apolipoprotein B-containing lipoprotein particles further reduce ASCVD risk, with a benefit proportional to the absolute achieved reduction in LDL-C without a lower limit^2–4^.

However, concerns regarding adverse effects, not limited to proven ones but also controversial ones^5^, are frequent causes of non-compliance to statin therapy. Among the controversial ones, concerns have been raised on cognitive function because the brain is a cholesterol-rich organ, and cholesterol is a major constituent of the myelin sheath^6^. To our knowledge, few studies have specifically explored the association between absolute LDL-C level achieved by statin use and the risk of cognitive dysfunction.

The results of epidemiological studies on the association between cholesterol levels and the risk of dementia, including Alzheimer’s disease (AD) and vascular dementia (VD), have been conflicting^7–12^, failing to address the interaction between such an association and the presence of diabetes or the use of statins. Although high total cholesterol (TC) in late life has been suggested as protective against dementia^10^, and a weaker association between LDL-C level and dementia risk has been reported in older people (aged 65 years or above) compared with those aged less than 65 years^12^, the previous studies did not specifically examine whether such trends are valid in the association between absolute cholesterol levels achieved by statin therapy and the risk of dementia^7–12^. Since statin affects cholesterol levels potently, lipid levels in statin users and non-users would be markedly varied. Also, vascular risk profiles, closely related to the risk of dementia^13,14^, would be highly variable between statin users and non-users in the real-world since statins are more widely and actively prescribed to individuals at higher vascular risk. In addition, most previous studies did not explore whether the presence of diabetes could modify the association between LDL-C level and dementia risk. Given that diabetes is a profound modifier of lipid profiles^15,16^, a major risk factor for dementia^13,14,17,18^, and one of the most important indications for intensive statin therapy^1^, the discrepancy among previous epidemiological studies needs to be resolved by large-scale population-based research offering a stratified analysis according to the presence of diabetes and the use of statin.

Therefore, we explored trends in the association between baseline cholesterol levels and the risk of dementia according to the presence of diabetes and the use of statin.

## Results

### Baseline characteristics

**Figure 1:**
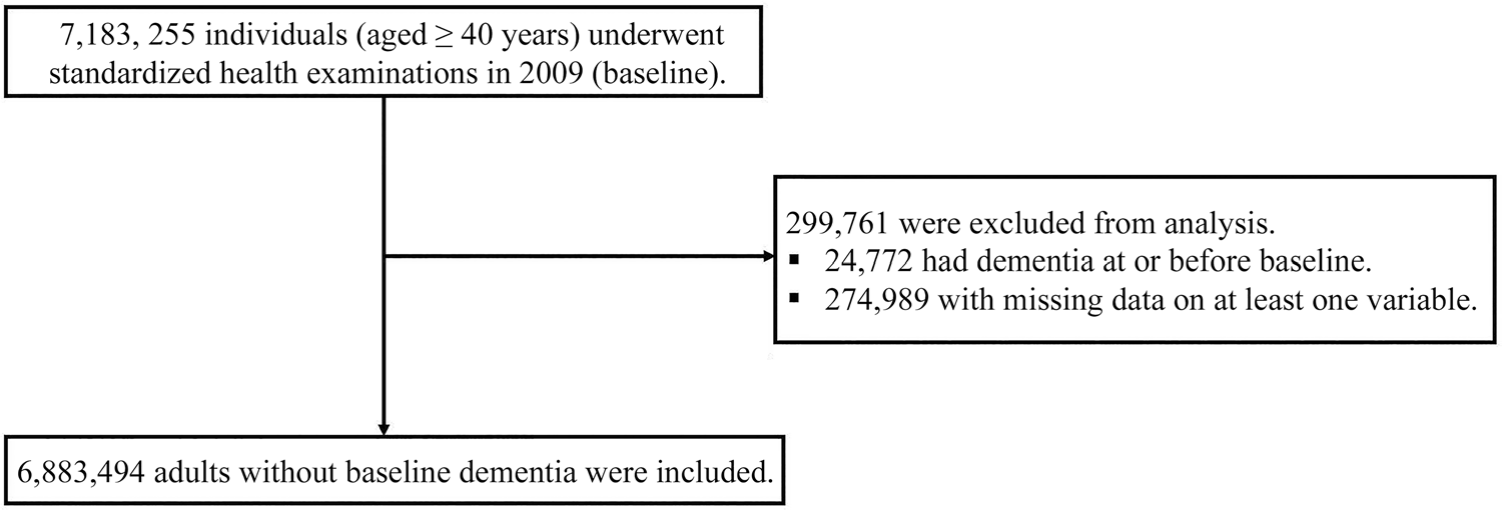
Flowchart outlining selection of the study population.

A total of 6,883,494 individuals was included (Fig. 1). Individuals with LDL-C in the lower quartiles were more likely to be male, current smokers, and heavy drinkers, and to have higher baseline estimated glomerular filtration rate (eGFR), MI, and stroke prevalence (Table 1). With increasing quartiles of LDL-C, an increasing trend was observed in body mass index (BMI) and proportion of nondrinkers.

**Table 1:** Baseline characteristics according to quartile of low-density lipoprotein cholesterol level.

LDL-C quartile^a^	Q1	Q2	Q3	Q4	p-value
N	1,716,390	1,754,964	1,705,307	1,706,833	
Age (years)	54.18 ± 10.85	53.77 ± 10.54	54.21 ± 10.30	55.25 ± 10.08	< 0.0001
**Age group [n (%)]**					< 0.0001
40–49 years	705,863 (41.12)	732,753 (41.75)	654,268 (38.37)	547,391 (32.07)	
50–59 years	474,681(27.66)	515,717 (29.39)	515,717 (29.39)	604,004 (35.39)	
60–69 years	337,639 (19.67)	324,201 (18.47)	330,805 (19.4)	369,539 (21.65)	
≥ 70 years	198,207 (11.55)	182,293 (10.39)	174,752 (10.25)	185,899 (10.89)	
Male sex [n (%)]	968,204 (56.41)	898,167 (51.18)	848,279 (49.74)	765,580 (44.85)	< 0.0001
BMI (kg/m_2_)	23.71 ± 3.12	23.77 ± 3.01	24.04 ± 2.97	24.39 ± 2.94	< 0.0001
SBP (mmHg)	124.27 ± 15.64	123.44 ± 15.32	124.05 ± 15.24	125.19 ± 15.41	< 0.0001
DBP (mmHg)	77.10 ± 10.30	76.75 ± 10.11	77.15 ± 10.07	77.80 ± 10.08	< 0.0001
Current smoker [n (%)]	419,102 (24.42)	364,174 (20.75)	341,647 (20.03)	323,736 (18.97)	< 0.0001
**Alcohol consumption [n (%)]**					< 0.0001
Nondrinkers (< 1 g/day)	907,188 (52.85)	1,007,182 (57.39)	1,013,450 (59.43)	1,090,459 (63.89)	
Moderate drinkers	653,500 (38.07)	639,773 (36.46)	600,173 (35.19)	540,400 (31.66)	
Heavy drinkers (≥ 30 g/day)	155,702 (9.07)	108,009 (6.15)	91,684 (5.38)	75,974 (4.45)	
Regular exercise [n (%)]	854,519 (49.79)	885,526 (50.46)	859,533 (50.40)	835,759 (48.97)	< 0.0001
Low household income (lowest 25%) [n (%)]	470,425 (27.41)	470,309 (26.80)	450,701 (26.43)	455,916 (26.71)	< 0.0001
Fasting glucose (mg/dl)	101.23 ± 27.62	98.75 ± 23.6	99.11 ± 23.23	100.72 ± 24.85	< 0.0001
Triglycerides (mg/dl)	127.84 (127.71–127.97)	112.66 (112.57–112.75)	115.04 (114.95–115.12)	121.81 (121.72–121.89)	< 0.0001
HDL-C (mg/dl)	55.73 ± 22.70	55.16 ± 20.71	54.75 ± 21.42	54.91 ± 24.02	< 0.0001
LDL-C (mg/dl)	74.28 ± 16.77	104.78 ± 6.30	126.4 ± 6.57	164.38 ± 51.24	< 0.0001
eGFR (ml/min/1.73 m^2^)	86.27 ± 35.59	85.40 ± 36.65	84.44 ± 37.38	83.25 ± 37.89	< 0.0001
Diabetes [n (%)]	275,743 (16.07)	189,498 (10.80)	169,484 (9.94)	177,146 (10.38)	< 0.0001
Hypertension [n (%)]	681,650 (39.71)	589,767 (33.61)	574,166 (33.67)	607,535 (35.59)	< 0.0001
Statin use [n (%)]	300,465 (17.51)	147,115 (8.38)	106,860 (6.27)	152,125 (8.91)	< 0.0001
Myocardial infarction [n (%)]	16,497 (0.96)	8,059 (0.46)	5,903 (0.35)	5,549 (0.33)	< 0.0001
Stroke [n (%)]	49,087 (2.86)	35,494 (2.02)	31,594 (1.85)	31,426 (1.84)	< 0.0001
Charlson Comorbidity Index	1.93 ± 1.50	1.93 ± 1.50	1.93 ± 1.50	1.93 ± 1.50	0.1180

Values are presented as number (%), mean ± standard deviation, or geometric mean (95% confidence interval).

*LDL-C* low-density lipoprotein cholesterol, *BMI* body mass index, *SBP* systolic blood pressure, *DBP* diastolic blood pressure, *HDL-C* high-density lipoprotein cholesterol, *eGFR* estimated glomerular filtration rate.

^a^LDL-C quartile ranges: Q1 (LDL-C < 94 mg/dl), Q2 (94 mg/dl ≤ LDL-C < 116 mg/dl), Q3 (116 mg/dl ≤ LDL-C < 139 mg/dl), Q4 (LDL-C ≥ 139 mg/dl).

### Incidence of dementia in the general population according to lipid parameters

During 55,849,826.75 person-years (median 8.33 years), 263,185 dementia cases were detected. In the general population, the hazards for all-cause dementia and AD were highest in the lowest quartiles (Q1) of LDL-C and high-density lipoprotein-cholesterol (HDL-C) (Table 2, Supplementary Table S1). When we used quintiles instead of quartiles for lipid stratifications and set the third quintile as the reference, the highest hazard of all-cause dementia was noted also in the lowest quintiles of LDL-C and HDL-C levels, demonstrating an inverted J-shaped relationship between quintiles of LDL-C or HDL-C and the hazard of all-cause dementia (Supplementary Table S2). With respect to VD, although a similar trend was observed in models 1 and 2, the Q2 and Q4 of LDL-C levels did not show a significantly lower hazard of VD compared to the Q1 of LDL-C level in model 4. Conversely, significantly higher hazards of all-cause dementia, AD, and VD were seen in higher triglycerides (TG) quartiles compared to the Q1 of TG. When the association between TC quartile and outcome incidence was assessed using Q1 as a reference, Q2 showed lower hazards of all-cause dementia, AD, and VD; and Q3 demonstrated lower hazards of all-cause dementia and AD. Compared to the Q1 of TC, Q4 did not exhibit significantly different hazards of all-cause dementia, AD, or VD, and Q3 did not differ in terms of the hazard of VD.

**Table 2:** Hazard ratios for the incidence of all-cause dementia according to quartile of lipid parameters.

Quartile of lipid parameters^a^	n	Events (n)	Follow-up duration (person-years)	Incidence rate (per 1000 person-years)	Hazard ratio (95% confidence interval)				
					Model 1	Model 2	Model 3	Model 3–1	Model 4
**LDL-C**									
Q1	1,716,390	70,173	13,812,376.82	5.08044	1 (Ref.)	1 (Ref.)	1 (Ref.)	1(Ref.)	1(Ref.)
Q2	1,754,964	62,949	14,259,651.33	4.41448	0.867 (0.858, 0.876)	0.906 (0.897, 0.916)	0.942 (0.932, 0.953)	0.942 (0.932, 0.953)	0.956 (0.945, 0.966)
Q3	1,705,307	60,825	13,890,566.36	4.37887	0.859 (0.850, 0.869)	0.882 (0.872, 0.891)	0.928 (0.918, 0.938)	0.928 (0.917, 0.938)	0.945 (0.935, 0.956)
Q4	1,706,833	69,238	13,887,232.24	4.98573	0.978 (0.968, 0.988)	0.917 (0.907, 0.927)	0.969 (0.959, 0.980)	0.969 (0.959, 0.980)	0.987 (0.976, 0.998)
**HDL-C**									
Q1	1,701,108	76,442	13,705,008.76	5.57767	1 (Ref.)	1 (Ref.)	1 (Ref.)	1(Ref.)	1(Ref.)
Q2	1,697,679	65,701	13,787,613.74	4.76522	0.853 (0.845, 0.862)	0.939 (0.929, 0.949)	0.960 (0.950, 0.970)	0.960 (0.950, 0.970)	0.958 (0.948, 0.968)
Q3	1,782,263	63,076	14,506,544.69	4.34811	0.779 (0.771, 0.787)	0.921 (0.911, 0.931)	0.949 (0.939, 0.959)	0.949 (0.939, 0.959)	0.946 (0.936, 0.956)
Q4	1,702,444	57,966	13,850,659.56	4.18507	0.751 (0.743, 0.759)	0.933 (0.923, 0.943)	0.965 (0.954, 0.975)	0.964 (0.954, 0.975)	0.961 (0.950, 0.971)
**Triglycerides**									
Q1	1,698,765	48,512	13,855,868.15	3.50119	1 (Ref.)	1 (Ref.)	1 (Ref.)	1(Ref.)	1(Ref.)
Q2	1,732,804	68,053	14,045,373.48	4.84523	1.384 (1.368, 1.400)	1.037 (1.025, 1.050)	1.027 (1.015, 1.039)	1.027 (1.015, 1.040)	1.027 (1.015, 1.039)
Q3	1,727,849	76,121	13,977,984.87	5.44578	1.556 (1.538, 1.573)	1.068 (1.056, 1.080)	1.048 (1.036, 1.061)	1.049 (1.036, 1.061)	1.048 (1.036, 1.060)
Q4	1,724,076	70,499	13,970,600.24	5.04624	1.442 (1.425, 1.459)	1.145 (1.132, 1.158)	1.096 (1.083, 1.109)	1.096 (1.083, 1.109)	1.096 (1.083, 1.109)
**Total cholesterol**									
Q1	1,720,451	71,228	13,836,389.14	5.14787	1 (Ref.)	1 (Ref.)	1 (Ref.)	1(Ref.)	1(Ref.)
Q2	1,713,211	61,467	13,928,119.96	4.41316	0.856 (0.846, 0.865)	0.924 (0.914, 0.934)	0.955 (0.944, 0.965)	0.955 (0.944, 0.965)	0.967 (0.957, 0.978)
Q3	1,710,041	60,823	13,933,296.61	4.36530	0.846 (0.837, 0.855)	0.905 (0.895, 0.915)	0.944 (0.934, 0.955)	0.944 (0.934, 0.955)	0.961 (0.950, 0.971)
Q4	1,739,791	69,667	14,152,021.04	4.92276	0.954 (0.944, 0.964)	0.952 (0.942, 0.962)	0.994 (0.984, 1.005)	0.994 (0.983, 1.005)	1.010 (0.999, 1.021)

Model 1: unadjusted.

Model 2: adjusted for age and sex.

Model 3: model 2 plus body mass index, diabetes, hypertension, current smoking status, alcohol consumption status, regular exercise, and estimated glomerular filtration rate.

Model 3–1: model 3 plus monthly household income, and Charlson Comorbidity Index.

Model 4: model 3 plus statin use.

*LDL-C* low-density lipoprotein cholesterol, *HDL-C* high-density lipoprotein cholesterol.

^a^LDL-C quartile ranges: Q1 (LDL-C < 94 mg/dl), Q2 (94 mg/dl ≤ LDL-C < 116 mg/dl), Q3 (116 mg/dl ≤ LDL-C < 139 mg/dl), Q4 (LDL-C ≥ 139 mg/dl); HDL-C quartiles: Q1 (HDL-C < 45 mg/dl), Q2 (45 mg/dl ≤ HDL-C < 53 mg/dl), Q3 (53 mg/dl ≤ HDL-C < 63 mg/dl), Q4 (HDL-C ≥ 63 mg/dl); triglyceride quartiles: Q1 (triglyceride < 80 mg/dl), Q2 (80 mg/dl ≤ triglyceride < 115 mg/dl), Q3 (115 mg/dl ≤ triglyceride < 169 mg/dl), Q4 ( triglyceride ≥ 169 mg/dl); and total cholesterol quartiles: Q1 (total cholesterol < 174 mg/dl), Q2 (174 mg/dl ≤ total cholesterol < 197 mg/dl), Q3 (197 mg/dl ≤ total cholesterol < 222 mg/dl), Q4 (total cholesterol ≥ 222 mg/dl).

### Incidence of dementia according to LDL-C level in individuals categorized by the presence of diabetes and statin use

**Figure 2:**
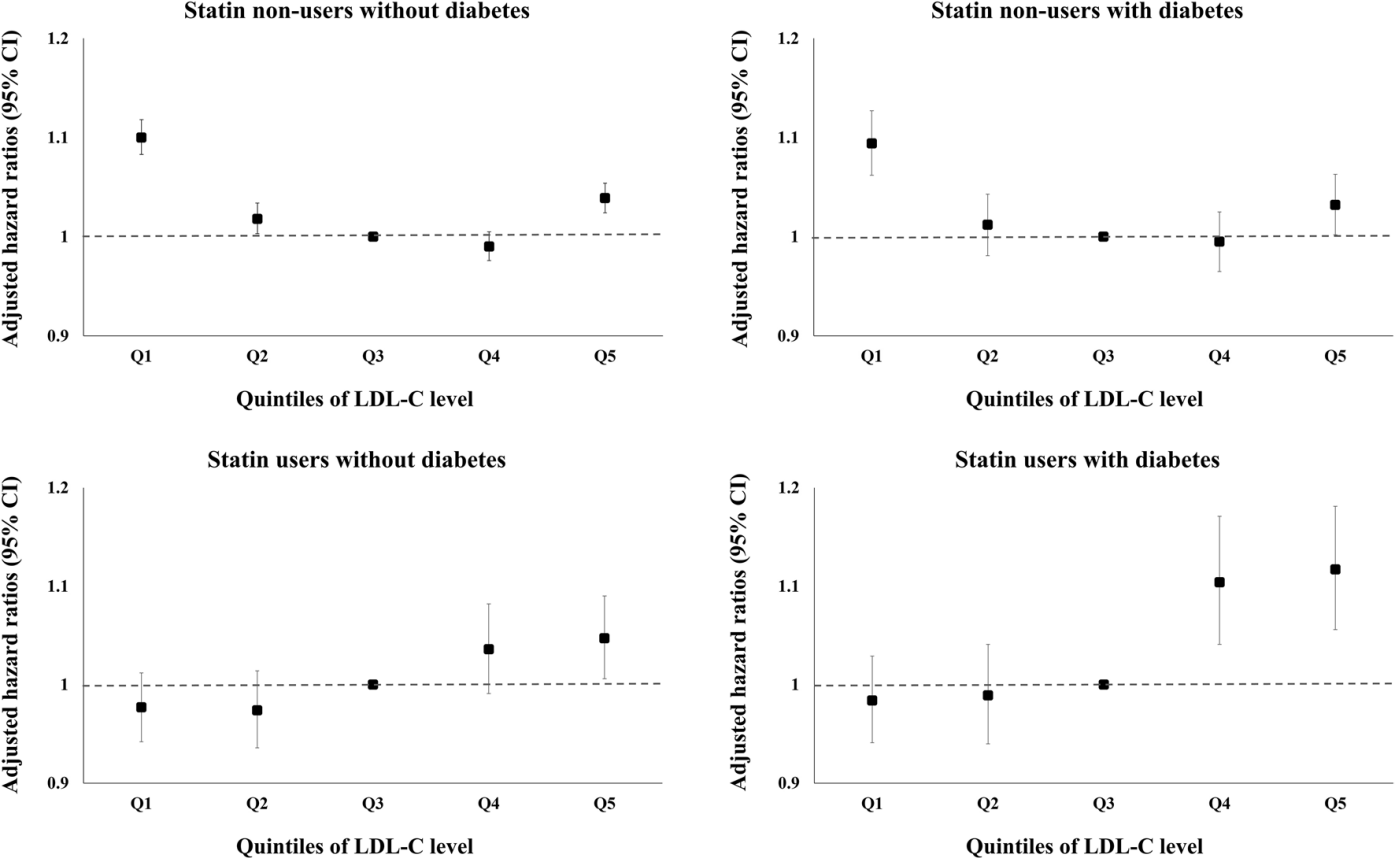
Hazard ratios for the incidence of all-cause dementia according to quintiles of low-density lipoprotein cholesterol levels in groups stratified according to the presence of diabetes and statin use**.** Adjusted for age, sex, body mass index, hypertension, current smoking status, alcohol consumption status, regular exercise, and estimated glomerular filtration rate. *LDL-C quintile ranges: Q1 (LDL-C < 89 mg/dl), Q2 (89 mg/dl ≤ LDL-C < 107 mg/dl), Q3 (reference, 107 mg/dl ≤ LDL-C < 124 mg/dl), Q4 (124 mg/dl ≤ LDL-C < 145 mg/dl), Q5 (LDL-C ≥ 145 mg/dl). *LDL-C* low-density lipoprotein cholesterol, *CI* confidence interval.

Consistent to previous reports that indicated diabetes as a major risk factor for dementia^13,14,17,18^, also in our dataset, individuals with diabetes exhibited a significantly higher hazard of all-cause dementia compared to those without (Supplementary Table S3), which provided one of the rationales for these stratified analyses. In statin non-users with or without diabetes, the hazards of all-cause dementia and AD were highest in the lowest quartile or quintile of LDL-C, exhibiting an inverted J-shaped relationship (Fig. 2, Supplementary Table S4). However, among statin users with or without diabetes, an advance in LDL-C quartile or quintile was associated with an increase in the hazard of all-cause dementia (hazard ratio, HR [95% confidence interval, CI] 1.010 [0.979–1.041] in Q2, 1.034 [1.000–1.070] in Q3, and 1.075 [1.042–1.108] in Q4 for those without diabetes; 1.015 [0.978–1.052] in Q2, 1.073 [1.028–1.121] in Q3, and 1.148 [1.103–1.196] in Q4 for those with diabetes) (Fig. 2, Supplementary Table S4). Additional adjustment for monthly household income and Charlson Comorbidity Index (CCI) demonstrated consistent findings with respect to the hazard of all-cause dementia. Regarding the hazard of AD, among statin users with or without diabetes, increasing trends of hazard according to the advance in LDL-C quartiles were shown after additional adjustment for these two factors.

### Incidence of dementia according to deciles of LDL-C level in statin users with diabetes

**Figure 3:**
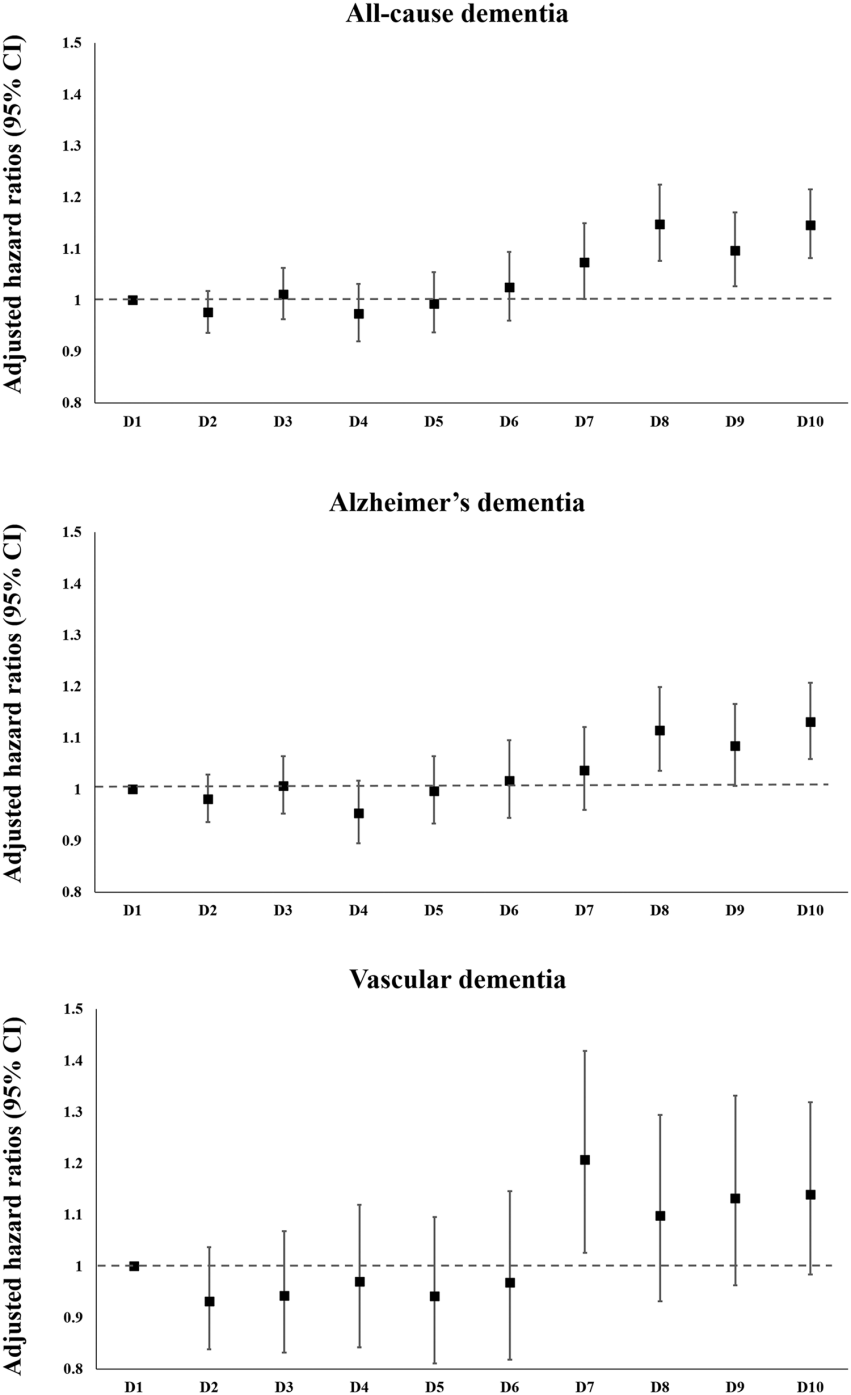
Hazard ratios for the incidence of all-cause dementia, Alzheimer’s disease, and vascular dementia among statin users with diabetes according to deciles of low-density lipoprotein cholesterol level. Adjusted for age, sex, body mass index, hypertension, current smoking status, alcohol consumption status, regular exercise, and estimated glomerular filtration rate. *LDL-C decile ranges: D1 (LDL-C < 75 mg/dl), D2 (75 mg/dl ≤ LDL-C < 89 mg/dl), D3 (89 mg/dl ≤ LDL-C < 99 mg/dl), D4 (99 mg/dl ≤ LDL-C < 107 mg/dl), D5 (107 mg/dl ≤ LDL-C < 116 mg/dl), D6 (116 mg/dl ≤ LDL-C < 124 mg/dl), D7 (124 mg/dl ≤ LDL-C < 133 mg/dl), D8 (133 mg/dl ≤ LDL-C < 145 mg/dl), D9 (145 mg/dl ≤ LDL-C < 162 mg/dl), D10 (LDL-C ≥ 162 mg/dl). *LDL-C* low-density lipoprotein cholesterol, *CI* confidence interval.

In subjects with diabetes using statins, the hazards of the outcomes were compared according to decile of LDL-C level to examine whether very low LDL-C level is associated with increased risk for dementia (Fig. 3). In statin users with diabetes, compared to the first decile group (D1, LDL-C < 75 mg/dl), the three highest decile groups (D8–D10, 133 mg/dl ≤ LDL-C) had a higher hazard of AD, and the highest four decile groups (D7–D10, 124 mg/dl ≤ LDL-C) presented a higher hazard of all-cause dementia.

Similar trends were observed in statin users with ASCVD (Supplementary Fig. S1), which is another indication for intensive statin therapy, and in statin users aged ≥ 70 years, the age population in which the protective effect of high cholesterol levels was originally suggested^10^ (Supplementary Table S5).

### Sensitivity analyses

Additional sensitivity analyses stratified by the statin use and restricting the subjects to individuals aged 40–60 years and to those aged ≥ 60 years demonstrated consistent results (Supplementary Tables S6 and S7). Sensitivity analyses in subpopulations categorized by the exposure duration to statins (never-users, irregular or short-term users, and continuous-users of statins) that accounted for the exposure duration to statins more in detail also yielded consistent findings, varied patterns of association according to statin exposure (Supplementary Table S8).

## Discussion

In this study, the presence of diabetes did not affect trends in the association between LDL-C level and the risk of dementia. In statin non-users, an inverted J-shaped relation was noted between LDL-C level and dementia risk, showing a seemingly paradoxical increase in dementia risk in lower LDL-C levels, regardless of the presence of diabetes. However, this trend was not observed in statin users regardless of the presence of diabetes, with an increasing trend in the hazards of all-cause dementia according to increasing LDL-C quartile or quintile. Additional analyses conducted among never-users and continuous-users of statins also demonstrated consistent findings. In statin users with diabetes, the group with LDL-C level in the lowest decile (LDL-C < 75 mg/dl) did not present increased risk of all-cause dementia. Rather, those with LDL-C level ≥ 124 mg/dl showed increased risk of all-cause dementia compared to the lowest decile (LDL-C < 75 mg/dl).

In this study, the risk of all-cause dementia in the general population and in statin non-users was increased in those with LDL-C level in both the lowest and the highest quintiles, showing an inverted J-shaped relationship. However, the trend was not observed in the association between absolute cholesterol levels achieved by statins and the risk of dementia. As in other studies showing the ‘cholesterol paradox’ in various diseases^19,20^, an increase in the risk of dementia in statin non-users with LDL-C level in the lowest quintile does not indicate a causative role of low LDL-C level in dementia etiology but might represent secondary factors such as chronic inflammation, which results in decreased TC, LDL-C, and HDL-C levels and increased TG level^19,21^. Weight loss and an accompanying decrease in cholesterol levels are often observed alongside cognitive decline shortly before the diagnosis of dementia, which also could have affected the trends observed herein. Furthermore, considering that individuals in lower LDL-C quartiles were more likely to be current smokers and heavy drinkers and had a higher prevalence of ASCVD, the effects of these potential confounders might remain despite adjustment. On the other hand, the increased risk of all-cause dementia in those with LDL-C level in the highest quintile in the general population, which was consistent regardless of the statin use (Fig. 2), could be explained by an increasingly accepted notion that atherosclerosis is a key aspect in AD as well as VD^13^. A recent meta-analysis also suggested that high cholesterol values may play a role in the development of AD^22^.

It is reassuring that the lack of increase in the risk of dementia with LDL-C level in the lowest quartile or quintile was consistently observed in statin users, also in those with diabetes. In those with diabetes, moderate- or high-intensity statins would have been more frequently used than in the general population because recent guidelines have recommended statin therapy with adequate intensity regardless of baseline LDL-C level in people with diabetes^1^. In this study, statin users with diabetes and LDL-C level in the lowest decile (LDL-C < 75 mg/dl) did not present increased risks of all-cause dementia. A similar trend was observed in statin users with ASCVD, which is another indication for intensive statin therapy^1^; statin users in their midlife (40–60 years) or late-life (≥ 60 years); and especially in statin users aged ≥ 70 years, the age population in which the protective effect of high cholesterol levels was originally suggested^10^. The hypothesis that extremely low blood cholesterol levels can impair neuronal homeostasis has a caveat because cerebral cholesterol is primarily produced locally^23^, and cerebral cholesterol levels may be independent of plasma levels^24^. Indeed, a recent Mendelian randomization study in those with proprotein convertase subtilisin-kexin type 9 (PCSK9) and 3-hydroxy-3-methylglutaryl-CoA reductase genetic variations^25^ and recent randomized controlled trials of ezetimibe and anti-PCSK9 monoclonal antibodies indicated that very low LDL-C level does not increase the incidence of neurocognitive adverse events^2,26^. Therefore, the paradoxically increased hazards observed with LDL-C level in the lowest quartile or quintile among statin non-users with diabetes are not likely to be clinically relevant and at least should not be extrapolated to statin users with diabetes who achieved low LDL-C level with statin therapy.

The strength of this study was the large number of subjects (N = 6,883,494), representing the entire Korean population. The Korean National Health Insurance System (KNHIS) not only covers the entire Korean population, but also has strict reimbursement criteria that mandate documentation of evidence for cognitive dysfunction (assessed by the Mini-Mental State Exam [MMSE] and either the Clinical Dementia Rating or Global Deterioration Scale [GDS]) to prescribe anti-dementia drugs, which is highly likely to prevent misclassification or over-diagnosis of dementia. Although diverse variables including lifestyle, anthropometric, and laboratory measures were collected in this large population, only 3.8% of the eligible subjects were excluded for having missing values on at least one variable. The sufficient power enabled stratified analyses according to the presence of diabetes and statin use and provided insights on the specific association between absolute LDL-C level achieved by statin use and the risk of dementia.

The limitations of this study should be addressed. First, the study population was comprised of a single ethnicity, and extrapolation of the results to other ethnicities should be cautious. Second, the definition of statin use versus non-use was based on prescription records, which might be different from actual drug use. However, there have been reports on the correlation between prescriptions and real exposure to medications^27,28^. In Korea, a prescription by physicians is necessary to obtain statins, and a simple refill at pharmacies is prohibited. Third, analyses were based on lipid measures at a single timepoint (baseline). Repeated measurements and varied levels during follow-up were not reflected due to the data unavailability in most of the individuals. However, previous studies on the association between lipid levels and adverse outcomes also used a single baseline lipid level to produce meaningful findings^29–34^. Fourth, the diagnosis of dementia was based on the records of diagnostic codes and prescriptions, and brain imaging or in-depth cognitive tests were not used directly. However, to minimize the misclassification or over-diagnosis, we used the information on the prescription of anti-dementia medications as well as diagnostic codes. Considering the strict reimbursement criteria in Korea that require the documentation of evidence for cognitive dysfunction (assessed by MMSE and either Clinical Dementia Rating or GDS) to prescribe anti-dementia drugs, it is less likely that other conditions may have been misclassified or over-diagnosed as dementia. Fifth, data on factors that may have significant impact on dementia risk, such as educational attainment and/or baseline cognitive functions were unavailable. However, although we could not directly adjust for the educational attainment because of the data unavailability, additional adjustment for income, which are closely related to educational level^35^, demonstrated consistent findings. Lastly, we could not fully reflect the exact dosage or intensity of statins due to data unavailability.

Although there was a seemingly paradoxical association between low LDL-C level and dementia risk in statin non-users, such a trend was not observed in statin users and is not likely to be clinically relevant. Rather, an increase in LDL-C level was associated with an increase in the risk of all-cause dementia in statin users. This trend was consistent regardless of the presence of diabetes, also in people with diabetes who achieved very low LDL-C level with statin therapy.

## Methods

### Data sources

For this nationwide, longitudinal, population-based cohort study, we used the KNHIS datasets from January 2002 to December 2017. The KNHIS is a compulsory health insurance system for all citizens operated by the Korean government and recommends standardized health examinations at least every two years through the national health screening program^36^. The KNHIS provides a public database containing the health information of > 50 million people, including a qualification database (containing information regarding sex, age, household income, residential area, and types of qualification), claims data (diagnoses defined by International Classification of Diseases [ICD] codes and prescriptions), and death information^36^. In addition, results of biennial standardized health examinations promoted by the KNHIS through national health screening program are also assembled into the database. The protocol for this study was approved by the Institutional Review Board (IRB) of Samsung Medical Center (no. 2020-09-088), and all methods were performed in accordance with the relevant guidelines and regulations. An informed consent exemption was granted by the IRB of Samsung Medical Center because the KNHIS provided the researchers with de-identified data.

### Study population

We included individuals aged ≥ 40 years who underwent health examinations between January and December 2009. The time point of the examination in 2009 was considered the baseline. We selected the year 2009 since lipid profiles including LDL-C, HDL-C, and TG were first introduced as components of standardized health examinations in 2009. In a total of 7,183,255 subjects, those with dementia at or before baseline (n = 24,772) and those with missing data for one or more variables (n = 274,989) were excluded (Fig. 1). Finally, 6,883,494 subjects were selected and followed from baseline until death, dementia diagnosis, or December 31, 2017, whichever came first.

### Measurements and definitions

Standardized health examinations are conducted only in hospitals certified by the KNHIS. All health examination institutions undergo regular quality assessments according to the Basic Act on National Health Examination in Korea. Blood tests, including plasma glucose, TG, HDL-C, and LDL-C, were performed after an overnight fast. The eGFR was calculated by the Modification of Diet in Renal Disease Study equation^37^. BMI (kg/m^2^) was calculated by dividing body weight (kg) by height squared (m^2^). Blood pressure was measured by qualified medical personnel using sphygmomanometers or oscillometric devices at brachial levels after the examinee rested in a sitting position for at least 5 min. Questionnaires were conducted regarding smoking status, alcohol consumption history, and physical activity. Participants were classified into nondrinkers, moderate drinkers, and heavy drinkers according to alcohol consumption status. Individuals with an average alcohol intake < 1 g/day were considered as nondrinkers, while average alcohol ingestion ≥ 30 g/day was defined as heavy consumption^38^. Regular exercise was classified as high-intensity physical activity causing extreme shortness of breath for > 20 min per session ≥ 3 days per week or moderate-intensity physical activity causing substantial shortness of breath for > 30 min per session ≥ 5 days per week^39^.

The presence of diabetes mellitus was defined as either (1) at least one claim per year under ICD-10 codes E10-14 and at least one claim per year for prescription of anti-diabetes medication or (2) fasting glucose level ≥ 126 mg/dl. The presence of hypertension was defined as one or more claims per year under ICD-10 codes I10 or I11 and at least one claim per year for the prescription of antihypertensive agents, or systolic/diastolic blood pressure ≥ 140/90 mmHg^40^. Myocardial infarction (MI) was defined as one or more claims under ICD-10 codes I21–I22 during hospitalization or at least two claims under these codes^40^; stroke was determined as recording of ICD-10 codes I63–I64 during hospitalization with claims for brain computed tomography or magnetic resonance imaging^40^. CCI was defined according to established methods^41^ using previously provided diagnostic codes^42^. The participants were classified as statin users when they had been prescribed statins during the year before baseline. Those who had never been prescribed statins during the year before baseline were classified as statin non-users.

### Study outcomes

The endpoint was incident all-cause dementia, and additional analyses were conducted after restricting the endpoint to AD or VD. Dementia was defined as the prescription of one or more anti-dementia medications and the presence of a claim for AD (ICD-10 F00 or G30), VD (ICD-10 F01), or another form of dementia (ICD-10 F02, F03, G23.1, G31)^43^. Anti-dementia medications included rivastigmine, galantamine, memantine, and donepezil hydrochloride. In Korea, strict reimbursement criteria require documentation of a MMSE score ≤ 26 and a Clinical Dementia Rating ≥ 1 or GDS stage ≥ 3 to file expense claims for anti-dementia drug prescriptions^44^. Identification of dementia type was based on diagnostic codes. If the codes for both AD and VD were present, the main diagnosis was considered the final diagnosis. When both codes for AD and VD were recorded as an additional diagnosis, the main diagnosis at the following visit was used as the final diagnosis^43^.

### Statistical analyses

Subjects were stratified into quartiles of baseline lipid parameters. The baseline characteristics of the study population are presented according to quartile of LDL-C. Continuous variables with normal distributions are described as mean ± standard deviation, and those with non-normal distributions are described as geometric mean and 95% CI. Categorical variables are presented as frequency and percentage. The incidence rates of outcome (per 1000 person-years) were calculated as the number of incident cases divided by the total follow-up duration. Cox proportional hazards regression analysis was performed to evaluate the HRs and 95% CIs for the outcome incidence according to quartiles of lipid parameters. Model 1 was unadjusted, and model 2 was adjusted for age and sex. Model 3 was adjusted for age, sex, BMI, diabetes status, hypertension, current smoking, alcohol consumption, regular exercise, and eGFR. Model 3–1 was additionally adjusted for monthly household income and CCI in addition to the variables included in model 3. Model 4 was further adjusted for statin use in addition to the confounders in model 3. The proportional hazard assumptions of the Cox models were ensured by Schoenfeld residuals. We classified the study population according to the presence of diabetes and statin use. Stratified analyses according to statin use were performed in individuals with or without diabetes. To evaluate the effect of highest or lowest values of lipid parameters on outcome hazards, the main analyses were repeated using quintiles instead of quartiles for lipid stratifications and setting the third quintile as the reference. Among statin users with diabetes, the hazard of the outcome was calculated according to deciles of LDL-C levels, to examine the association between very low LDL-C level and the hazard of the outcome in individuals under intensive statin therapy due to high risk for subsequent ASCVD. P-values were considered significant at < 0.05, and all analyses were performed using SAS Version 9.4 (SAS Institute, Cary, NC, USA).

### Sensitivity analyses

We conducted sensitivity analyses stratified by the statin use and restricting the subjects to individuals aged 40–60 years and to those aged ≥ 60 years considering previous suggestion that midlife (40–60 years) and late‐life (≥ 60 years) exposure to high cholesterol levels may have varied relation to dementia risk^22^. To account for the exposure duration to statins more in detail, we investigated the association between LDL-C levels and the hazard of dementia according to the presence of diabetes in subpopulations categorized by the exposure duration to statins (never-users, irregular or short-term users, and continuous-users of statins). Never-users of statins were defined as individuals who had never been prescribed for statins from 2002 to baseline. Those who had been prescribed statins for < 180 days, and for ≥ 180 days during the year before baseline were defined as irregular or short-term users, and continuous-users, respectively.

## Supplementary Information

Supplementary Information.

## Data Availability

The data that support the findings of this study are available from the Korean National Health Insurance Service (KNHIS) but restrictions apply to the availability of these data, which were used under license for the current study, and so are not publicly available. Data are however available from the corresponding authors upon reasonable request and with permission of the KNHIS.

## Abbreviations

AD: Alzheimer’s disease
ASCVD: Atherosclerotic cardiovascular disease
BMI: Body mass index
CI: Confidence interval
eGFR: Estimated glomerular filtration rate
GDS: Global Deterioration Scale
HDL-C: High-density lipoprotein cholesterol
HR: Hazard ratio
ICD: International Classification of Diseases
IRB: Institutional Review Board
KNHIS: Korean National Health Insurance System
LDL-C: Low-density lipoprotein-cholesterol
MI: Myocardial infarction
MMSE: Mini-Mental State Exam
PCSK9: Proprotein convertase subtilisin-kexin type 9
TC: Total cholesterol
TG: Triglycerides
VD: Vascular dementia
