# Supplementary material for: Association between cholesterol levels and dementia risk according to the presence of diabetes and statin use: a nationwide cohort study
Source: Sci Rep. 2022 Nov 12;12:19383. doi: 10.1038/s41598-022-24153-1 (PMC9653412; doi:10.1038/s41598-022-24153-1)
Supplement: Supplementary file 1 — Supplementary Information. [file 41598_2022_24153_MOESM1_ESM.docx]

**Supplementary Table S1.**  Hazard ratios for the incidence of Alzheimer’s disease and vascular dementia according to quartiles of lipid parameters.

| **Quartile of lipid parameters^*^** | **n** | **Events (n)** | **Follow-up duration (person-years)** | **Incidence rate (per 1000 person-years)** | **Hazard ratio (95% confidence interval)** | | | | |
| --- | --- | --- | --- | --- | --- | --- | --- | --- | --- |
|  |  |  |  |  | **Model 1** | **Model 2** | **Model 3** | **Model 3-1** | **Model 4** |
| **Alzheimer’s disease** | | | | | | | | | |
| **LDL-C** | | | | | | | | | |
| Q1 | 1716390 | 55456 | 13812376.82 | 4.01495 | 1(Ref.) | 1(Ref.) | 1(Ref.) | 1(Ref.) | 1(Ref.) |
| Q2 | 1754964 | 50123 | 14259651.33 | 3.51502 | 0.873 (0.862, 0.883) | 0.910 (0.899, 0.921) | 0.942 (0.931, 0.954) | 0.942(0.931,0.954) | 0.956 (0.944, 0.968) |
| Q3 | 1705307 | 48510 | 13890566.36 | 3.49230 | 0.866 (0.856, 0.877) | 0.885 (0.875, 0.896) | 0.927 (0.916, 0.938) | 0.927(0.915,0.938) | 0.945 (0.933, 0.957) |
| Q4 | 1706833 | 55452 | 13887232.24 | 3.99302 | 0.989 (0.978, 1.001) | 0.921 (0.910, 0.932) | 0.969 (0.958, 0.981) | 0.969(0.957,0.981) | 0.987 (0.975, 0.999) |
| **HDL-C** | | | | | | | | | |
| Q1 | 1701108 | 60388 | 13705008.76 | 4.40627 | 1(Ref.) | 1(Ref.) | 1(Ref.) | 1(Ref.) | 1(Ref.) |
| Q2 | 1697679 | 52435 | 13787613.74 | 3.80305 | 0.862 (0.852, 0.872) | 0.948 (0.937, 0.959) | 0.966 (0.955, 0.978) | 0.966(0.955,0.978) | 0.965 (0.953, 0.976) |
| Q3 | 1782263 | 50316 | 14506544.69 | 3.46850 | 0.787 (0.777, 0.796) | 0.929 (0.918, 0.940) | 0.954 (0.943, 0.966) | 0.954(0.943,0.966) | 0.951 (0.940, 0.963) |
| Q4 | 1702444 | 46402 | 13850659.56 | 3.35017 | 0.761 (0.752, 0.770) | 0.945 (0.933, 0.956) | 0.972 (0.960, 0.984) | 0.972(0.96,0.984) | 0.968 (0.956, 0.980) |
| **Triglycerides** | | | | | | | | | |
| Q1 | 1698765 | 38926 | 13855868.15 | 2.80935 | 1(Ref.) | 1(Ref.) | 1(Ref.) | 1(Ref.) | 1(Ref.) |
| Q2 | 1732804 | 54461 | 14045373.48 | 3.87750 | 1.381 (1.363, 1.399) | 1.027 (1.013, 1.040) | 1.021 (1.008, 1.034) | 1.021(1.008,1.034) | 1.020 (1.007, 1.034) |
| Q3 | 1727849 | 60560 | 13977984.87 | 4.33253 | 1.543 (1.523, 1.562) | 1.048 (1.034, 1.061) | 1.036 (1.023, 1.049) | 1.036(1.023,1.050) | 1.035 (1.022, 1.049) |
| Q4 | 1724076 | 55594 | 13970600.24 | 3.97936 | 1.417 (1.399, 1.436) | 1.117 (1.103, 1.132) | 1.082 (1.068, 1.097) | 1.082(1.068,1.097) | 1.082 (1.068, 1.097) |
| **Total cholesterol** | | | | | | | | | |
| Q1 | 1720451 | 56450 | 13836389.14 | 4.07982 | 1(Ref.) | 1(Ref.) | 1(Ref.) | 1(Ref.) | 1(Ref.) |
| Q2 | 1713211 | 48986 | 13928119.96 | 3.51706 | 0.860 (0.850, 0.870) | 0.926 (0.915, 0.937) | 0.955 (0.943, 0.966) | 0.955(0.943,0.966) | 0.967 (0.955, 0.979) |
| Q3 | 1710041 | 48449 | 13933296.61 | 3.47721 | 0.850 (0.839, 0.860) | 0.904 (0.893, 0.915) | 0.942 (0.930, 0.953) | 0.941(0.930,0.953) | 0.958 (0.946, 0.970) |
| Q4 | 1739791 | 55656 | 14152021.04 | 3.93272 | 0.961 (0.950, 0.972) | 0.951 (0.940, 0.962) | 0.991 (0.980, 1.003) | 0.991(0.979,1.003) | 1.008 (0.995, 1.020) |
| **Vascular dementia** | | | | | | | | | |
| **LDL-C** | | | | | | | | | |
| Q1 | 1716390 | 10621 | 13812376.82 | 0.76895 | 1(Ref.) | 1(Ref.) | 1(Ref.) | 1(Ref.) | 1(Ref.) |
| Q2 | 1754964 | 9359 | 14259651.33 | 0.65633 | 0.852 (0.829, 0.876) | 0.900 (0.875, 0.925) | 0.955 (0.929, 0.982) | 0.955(0.929,0.982) | 0.972 (0.945,1.000) |
| Q3 | 1705307 | 8956 | 13890566.36 | 0.64475 | 0.836 (0.813, 0.860) | 0.872 (0.848, 0.897) | 0.939 (0.913, 0.966) | 0.939(0.913,0.966) | 0.962 (0.934, 0.990) |
| Q4 | 1706833 | 9946 | 13887232.24 | 0.71620 | 0.929 (0.904, 0.954) | 0.903 (0.879, 0.929) | 0.973 (0.946, 1.000) | 0.973(0.946,1.000) | 0.995 (0.967, 1.024) |
| **HDL-C** | | | | | | | | | |
| Q1 | 1701108 | 11614 | 13705008.76 | 0.84743 | 1(Ref.) | 1(Ref.) | 1(Ref.) | 1(Ref.) | 1(Ref.) |
| Q2 | 1697679 | 9738 | 13787613.74 | 0.70629 | 0.833 (0.811, 0.855) | 0.917 (0.893, 0.942) | 0.951 (0.926, 0.977) | 0.951(0.926,0.977) | 0.949 (0.924, 0.975) |
| Q3 | 1782263 | 9303 | 14506544.69 | 0.64130 | 0.756 (0.736, 0.777) | 0.895 (0.871, 0.920) | 0.945 (0.919, 0.971) | 0.945(0.919,0.971) | 0.942 (0.916, 0.968) |
| Q4 | 1702444 | 8227 | 13850659.56 | 0.59398 | 0.701 (0.681, 0.721) | 0.874 (0.850, 0.899) | 0.934 (0.907, 0.961) | 0.934(0.907,0.961) | 0.930 (0.903, 0.957) |
| **Triglycerides** | | | | | | | | | |
| Q1 | 1698765 | 6943 | 13855868.15 | 0.50109 | 1(Ref.) | 1(Ref.) | 1(Ref.) | 1(Ref.) | 1(Ref.) |
| Q2 | 1732804 | 9870 | 14045373.48 | 0.70272 | 1.402 (1.360, 1.446) | 1.092 (1.059, 1.126) | 1.053 (1.021, 1.086) | 1.053(1.021,1.086) | 1.052 (1.020, 1.085) |
| Q3 | 1727849 | 11213 | 13977984.87 | 0.80219 | 1.601 (1.554, 1.650) | 1.156 (1.121, 1.191) | 1.081 (1.049, 1.115) | 1.082(1.049,1.115) | 1.081 (1.048, 1.114) |
| Q4 | 1724076 | 10856 | 13970600.24 | 0.77706 | 1.551 (1.505, 1.598) | 1.268 (1.230, 1.307) | 1.128 (1.093, 1.163) | 1.128(1.093,1.164) | 1.128 (1.093, 1.163) |
| **Total cholesterol** | | | | | | | | | |
| Q1 | 1720451 | 10701 | 13836389.14 | 0.77340 | 1(Ref.) | 1(Ref.) | 1(Ref.) | 1(Ref.) | 1(Ref.) |
| Q2 | 1713211 | 8973 | 13928119.96 | 0.64424 | 0.832 (0.809, 0.855) | 0.905 (0.880, 0.931) | 0.944 (0.918, 0.971) | 0.944(0.918,0.971) | 0.960 (0.933, 0.987) |
| Q3 | 1710041 | 9100 | 13933296.61 | 0.65311 | 0.843 (0.819, 0.867) | 0.917 (0.892, 0.943) | 0.965 (0.938, 0.993) | 0.965(0.938,0.993) | 0.986 (0.958, 1.015) |
| Q4 | 1739791 | 10108 | 14152021.04 | 0.71424 | 0.922 (0.897, 0.947) | 0.954 (0.928, 0.981) | 0.998 (0.970, 1.026) | 0.997(0.970,1.026) | 1.018 (0.989, 1.047) |

^*^LDL-C quartile ranges: Q1 (LDL-C <94 mg/dl), Q2 (94 mg/dl ≤LDL-C <116 mg/dl), Q3 (116 mg/dl ≤LDL-C <139 mg/dl), Q4 (LDL-C ≥139 mg/dl); HDL-C quartiles: Q1 (HDL-C <45 mg/dl), Q2 (45 mg/dl ≤HDL-C <53 mg/dl), Q3 (53 mg/dl ≤HDL-C <63 mg/dl), Q4 (HDL-C ≥63 mg/dl); triglyceride quartiles: Q1 (triglyceride <80 mg/dl), Q2 (80 mg/dl ≤ triglyceride <115 mg/dl), Q3 (115 mg/dl ≤ triglyceride <169 mg/dl), Q4 (triglyceride ≥169 mg/dl); and total cholesterol quartiles: Q1 (total cholesterol <174 mg/dl), Q2 (174 mg/dl ≤ total cholesterol <197 mg/dl), Q3 (197 mg/dl ≤ total cholesterol <222 mg/dl), Q4 (total cholesterol ≥222 mg/dl).

Model 1: unadjusted.

Model 2: adjusted for age and sex.

Model 3: model 2 plus body mass index, diabetes, hypertension, current smoking status, alcohol consumption status, regular exercise, and estimated glomerular filtration rate.

Model 3-1: model 3 plus monthly household income, and Charlson Comorbidity Index.

Model 4: model 3 plus statin use.

LDL-C, low-density lipoprotein cholesterol; HDL-C, high-density lipoprotein cholesterol.

**Supplementary Table S2.** Hazard ratios for the incidence of all-cause dementia according to quintiles of lipid parameters.

| **Quintiles of lipid parameters^*^** | **n** | **Events (n)** | **Follow-up duration (person-years)** | **Incidence rate (per 1000 person-years)** | **Hazard ratio (95% confidence interval)** | | | |
| --- | --- | --- | --- | --- | --- | --- | --- | --- |
|  |  |  |  |  | **Model 1** | **Model 2** | **Model 3** | **Model 4** |
| **LDL-C** | | | | | | | | |
| Q1 | 1387825 | 58044 | 11149883.35 | 5.20579 | 1.203 (1.188,1.217) | 1.137 (1.124,1.151) | 1.081 (1.068,1.094) | 1.061 (1.048,1.074) |
| Q2 | 1334905 | 48498 | 10833356.74 | 4.47673 | 1.032 (1.019,1.045) | 1.030 (1.017,1.042) | 1.016 (1.004,1.029) | 1.011 (0.998,1.023) |
| Q3 | 1399669 | 49456 | 11390909.51 | 4.34171 | 1(Ref.) | 1(Ref.) | 1(Ref.) | 1(Ref.) |
| Q4 | 1393626 | 50674 | 11354083.31 | 4.46306 | 1.027 (1.015,1.040) | 0.989 (0.977,1.001) | 0.996 (0.984,1.009) | 0.999 (0.986,1.011) |
| Q5 | 1367469 | 56513 | 11121593.83 | 5.08138 | 1.169 (1.155,1.183) | 1.034 (1.022,1.047) | 1.044 (1.032,1.057) | 1.045 (1.033,1.058) |
| **HDL-C** | | | | | | | | |
| Q1 | 1327954 | 61273 | 10681386.24 | 5.73643 | 1.278 (1.263,1.293) | 1.093 (1.080,1.105) | 1.060 (1.048,1.072) | 1.063 (1.051,1.075) |
| Q2 | 1410552 | 55896 | 11443968.02 | 4.88432 | 1.087 (1.074,1.100) | 1.021 (1.008,1.033) | 1.011 (0.999,1.023) | 1.012 (1.000,1.024) |
| Q3 | 1448834 | 52945 | 11785460.47 | 4.4924 | 1(Ref.) | 1(Ref.) | 1(Ref.) | 1(Ref.) |
| Q4 | 1261766 | 44435 | 10270231.98 | 4.32658 | 0.963 (0.951,0.976) | 0.997 (0.984,1.010) | 1.002 (0.990,1.015) | 1.002 (0.989,1.014) |
| Q5 | 1434388 | 48636 | 11668780.03 | 4.16804 | 0.929 (0.918,0.940) | 1.013 (1.001,1.026) | 1.019 (1.007,1.032) | 1.017 (1.005,1.030) |
| **Triglycerides** | | | | | | | | |
| Q1 | 1388402 | 37996 | 11333205.51 | 3.35263 | 0.639 (0.630,0.647) | 0.951 (0.939,0.964) | 0.965 (0.952,0.977) | 0.965 (0.953,0.978) |
| Q2 | 1386261 | 51046 | 11250181.3 | 4.53735 | 0.864 (0.854,0.874) | 0.979 (0.967,0.990) | 0.986 (0.974,0.997) | 0.985 (0.974,0.997) |
| Q3 | 1363387 | 57932 | 11032839.02 | 5.25087 | 1(Ref.) | 1(Ref.) | 1(Ref.) | 1(Ref.) |
| Q4 | 1371448 | 60909 | 11097055.77 | 5.48875 | 1.045 (1.033,1.057) | 1.033 (1.022,1.045) | 1.024 (1.013,1.036) | 1.024 (1.013,1.036) |
| Q5 | 1373996 | 55302 | 11136545.15 | 4.96581 | 0.946 (0.935,0.957) | 1.109 (1.096,1.122) | 1.072 (1.060,1.085) | 1.073 (1.060,1.086) |
| **Total cholesterol** | | | | | | | | |
| Q1 | 1349004 | 57600 | 10823236.33 | 5.32188 | 1.206 (1.191,1.220) | 1.107 (1.094,1.120) | 1.058 (1.046,1.071) | 1.040 (1.027,1.053) |
| Q2 | 1381921 | 49637 | 11224235.16 | 4.42231 | 1.000 (0.987,1.012) | 1.010 (0.998,1.023) | 0.999 (0.987,1.012) | 0.995 (0.982,1.007) |
| Q3 | 1381843 | 49776 | 11248029.35 | 4.42531 | 1(Ref.) | 1(Ref.) | 1(Ref.) | 1(Ref.) |
| Q4 | 1391148 | 49641 | 11338356.92 | 4.37815 | 0.989 (0.977,1.001) | 0.986 (0.974,0.998) | 0.991 (0.979,1.003) | 0.993 (0.981,1.005) |
| Q5 | 1379578 | 56531 | 11215968.98 | 5.04022 | 1.139 (1.125,1.152) | 1.049 (1.037,1.062) | 1.053 (1.040,1.066) | 1.053 (1.041,1.066) |

^*^LDL-C quintile ranges: Q1 (LDL-C <89 mg/dl), Q2 (89 mg/dl ≤LDL-C <107 mg/dl), Q3 (107 mg/dl ≤LDL-C <124 mg/dl), Q4 (124 mg/dl ≤LDL-C <145 mg/dl), Q5 (LDL-C ≥145 mg/dl); HDL-C quintile ranges: Q1 (HDL-C <43 mg/dl), Q2 (43 mg/dl ≤HDL-C <50 mg/dl), Q3 (50 mg/dl ≤HDL-C <57 mg/dl), Q4 (57 mg/dl ≤HDL-C <65 mg/dl), Q5 (HDL-C ≥65 mg/dl); triglyceride quintile ranges: Q1 (triglyceride <74 mg/dl), Q2 (74 mg/dl ≤ triglyceride <101 mg/dl), Q3 (101 mg/dl ≤ triglyceride <133 mg/dl), Q4 (133 mg/dl ≤ triglyceride <187 mg/dl), Q5 (triglyceride ≥ 187 mg/dl); and total cholesterol quintile ranges: Q1 (total cholesterol <168 mg/dl), Q2 (168 mg/dl ≤ total cholesterol <188 mg/dl), Q3 (188 mg/dl ≤ total cholesterol <206 mg/dl), Q4 (206 mg/dl ≤ total cholesterol <229 mg/dl), Q5 (total cholesterol ≥229 mg/dl).

Model 1: unadjusted.

Model 2: adjusted for age and sex.

Model 3: model 2 plus body mass index, diabetes, hypertension, current smoking status, alcohol consumption status, regular exercise, and estimated glomerular filtration rate.

Model 4: model 3 plus statin use.

LDL-C, low-density lipoprotein cholesterol; HDL-C, high-density lipoprotein cholesterol.

**Supplementary Table S3.** Hazard ratios for the incidence of all-cause dementia according to the presence of diabetes.

| **Group** | **n** | **Events (n)** | **Follow-up duration (person-years)** | **Incidence rate (per 1000 person-years)** | **Hazard ratio (95% confidence interval)** | | |
| --- | --- | --- | --- | --- | --- | --- | --- |
|  |  |  |  |  | **Model 1** | **Model 2** | **Model 3** |
| Individuals without diabetes | 6071623 | 200036 | 49502467.07 | 4.04093 | 1(Ref.) | 1(Ref.) | 1(Ref.) |
| Individuals with diabetes | 811871 | 63149 | 6347359.68 | 9.94886 | 2.482 (2.460,2.505) | 1.492 (1.479,1.506) | 1.481 (1.468,1.495) |

Model 1: unadjusted.

Model 2: adjusted for age and sex.

Model 3: model 2 plus body mass index, hypertension, current smoking status, alcohol consumption status, regular exercise, and estimated glomerular filtration rate.

**Supplementary Table S4.**  Hazard ratios stratified according to statin use among individuals with or without diabetes for the incidence of all-cause dementia, Alzheimer’s disease, and vascular dementia according to quartiles of low-density lipoprotein cholesterol level.

| **LDL-C quartile^*^** | **All-cause dementia** | | **Alzheimer’s dementia** | | **Vascular dementia** | |
| --- | --- | --- | --- | --- | --- | --- |
|  | **Statin non-users** | **Statin users** | **Statin non-users** | **Statin users** | **Statin non-users** | **Statin users** |
|  | **HRs (95% CIs)** | | | | | |
| **Individuals without diabetes** | | | | | | |
| Adjusted for age, sex, BMI, hypertension, current smoking status, alcohol consumption status, regular exercise, and eGFR | | | | | | |
| Q1 | 1(Ref.) | 1(Ref.) | 1(Ref.) | 1(Ref.) | 1(Ref.) | 1(Ref.) |
| Q2 | 0.929 (0.916, 0.942) | 1.010 (0.979, 1.041) | 0.959 (0.947, 0.972) | 0.975 (0.944, 1.007) | 1.029 (1.015, 1.043) | 0.990 (0.954, 1.027) |
| Q3 | 0.911 (0.899, 0.924) | 1.034 (1.000, 1.070) | 0.951 (0.939, 0.964) | 0.940 (0.910, 0.971) | 1.048 (1.033, 1.063) | 1.043 (1.007, 1.081) |
| Q4 | 0.950 (0.937, 0.964) | 1.075 (1.042, 1.108) | 0.965 (0.952, 0.979) | 0.947 (0.916, 0.978) | 1.084 (1.069, 1.101) | 1.073 (1.035, 1.113) |
| Adjusted for age, sex, BMI, hypertension, current smoking status, alcohol consumption status, regular exercise, eGFR, monthly household income, and CCI | | | | | | |
| Q1 | 1(Ref.) | 1(Ref.) | 1(Ref.) | 1(Ref.) | 1(Ref.) | 1(Ref.) |
| Q2 | 0.929 (0.916, 0.942) | 1.010 (0.979, 1.041) | 0.930 (0.915, 0.945) | 1.020 (0.985, 1.055) | 0.956 (0.921, 0.992) | 0.990 (0.917, 1.070) |
| Q3 | 0.911 (0.899, 0.924) | 1.034 (0.999, 1.070) | 0.915 (0.900, 0.929) | 1.034 (0.995, 1.075) | 0.926 (0.892, 0.960) | 0.989 (0.906, 1.080) |
| Q4 | 0.951 (0.938, 0.964) | 1.074 (1.041, 1.107) | 0.954 (0.940, 0.969) | 1.081 (1.045, 1.119) | 0.956 (0.922, 0.992) | 0.975 (0.899, 1.057) |
| **Individuals with diabetes** | | | | | | |
| Adjusted for age, sex, BMI, hypertension, current smoking status, alcohol consumption status, regular exercise, and eGFR | | | | | | |
| Q1 | 1(Ref.) | 1(Ref.) | 1(Ref.) | 1(Ref.) | 1(Ref.) | 1(Ref.) |
| Q2 | 0.935 (0.910, 0.960) | 1.015 (0.978, 1.052) | 0.951 (0.928, 0.975) | 0.945 (0.911, 0.981) | 1.009 (0.977, 1.043) | 1.031 (0.982, 1.082) |
| Q3 | 0.925 (0.900, 0.950) | 1.073 (1.028, 1.121) | 0.941 (0.917, 0.966) | 0.943 (0.908, 0.980) | 1.017 (0.985, 1.049) | 1.035 (0.988, 1.085) |
| Q4 | 0.948 (0.923, 0.974) | 1.148 (1.103, 1.196) | 0.975 (0.949, 1.003) | 0.967 (0.928, 1.007) | 1.067 (1.035, 1.100) | 1.151 (1.099, 1.204) |
| Adjusted for age, sex, BMI, hypertension, current smoking status, alcohol consumption status, regular exercise, eGFR, monthly household income, and CCI | | | | | | |
| Q1 | 1(Ref.) | 1(Ref.) | 1(Ref.) | 1(Ref.) | 1(Ref.) | 1(Ref.) |
| Q2 | 0.935 (0.910, 0.960) | 1.014 (0.978, 1.052) | 0.930 (0.902, 0.959) | 1.013 (0.973, 1.056) | 0.975 (0.911, 1.043) | 0.959 (0.874, 1.054) |
| Q3 | 0.925 (0.900, 0.950) | 1.073 (1.028, 1.120) | 0.919 (0.891, 0.948) | 1.044 (0.994, 1.097) | 0.991 (0.925, 1.061) | 1.109 (0.996, 1.234) |
| Q4 | 0.948 (0.923, 0.974) | 1.148 (1.102, 1.195) | 0.939 (0.911, 0.968) | 1.136 (1.085, 1.189) | 1.033 (0.965, 1.106) | 1.157 (1.044, 1.282) |

^*^LDL-C quartile ranges: Q1 (LDL-C <94 mg/dl), Q2 (94 mg/dl ≤LDL-C <116 mg/dl), Q3 (116 mg/dl ≤LDL-C <139 mg/dl), Q4 (LDL-C ≥139 mg/dl).

Abbreviations: LDL-C, low-density lipoprotein cholesterol; HR, hazard ratio; CI, confidence interval; BMI, body mass index, eGFR, estimated glomerular filtration rate; CCI, Charlson Comorbidity Index.

**Supplementary Table S5.**  Hazard ratios stratified according to statin use for the incidence of all-cause dementia, Alzheimer’s disease, and vascular dementia according to low-density lipoprotein cholesterol level quartile among subjects aged ≥70 years.

| **LDL-C quartile^*^** | **Statin non-users** | | | | | | **Statin users** | | | | |
| --- | --- | --- | --- | --- | --- | --- | --- | --- | --- | --- | --- |
|  | **n** | | **Events (n)** | **Follow-up duration (person-years)** | **Incidence rate (per 1000 person-years)** | **HRs (95% CIs)** | **n** | **Events (n)** | **Follow-up duration (person-years)** | **Incidence rate (per 1000 person-years)** | **HRs (95% CIs)** |
| **All-cause dementia** | | | | | | | | | | | |
| Q1 | | 137100 | 29855 | 942039.93 | 31.6919 | 1(Ref.) | 61107 | 13607 | 434264.41 | 31.3334 | 1(Ref.) |
| Q2 | | 154304 | 33465 | 1093020.62 | 30.6170 | 0.952 (0.937, 0.967) | 27989 | 6374 | 200967.29 | 31.7166 | 1.019 (0.989, 1.050) |
| Q3 | | 156419 | 34128 | 1120847.15 | 30.4484 | 0.945 (0.930, 0.960) | 18333 | 4367 | 131454.82 | 33.2205 | 1.070 (1.034, 1.107) |
| Q4 | | 163386 | 37425 | 1172810.47 | 31.9105 | 0.974 (0.959, 0.989) | 22513 | 5645 | 160355.83 | 35.2030 | 1.128 (1.093, 1.164) |
| **Alzheimer’s disease** | | | | | | | | | | | |
| Q1 | | 137100 | 24242 | 942039.93 | 25.7335 | 1(Ref.) | 61107 | 10950 | 434264.41 | 25.2151 | 1(Ref.) |
| Q2 | | 154304 | 27255 | 1093020.62 | 24.9355 | 0.951 (0.934, 0.967) | 27989 | 5183 | 200967.29 | 25.7903 | 1.024 (0.990, 1.058) |
| Q3 | | 156419 | 27902 | 1120847.15 | 24.8937 | 0.945 (0.929, 0.962) | 18333 | 3531 | 131454.82 | 26.8609 | 1.068 (1.028, 1.109) |
| Q4 | | 163386 | 30672 | 1172810.47 | 26.1526 | 0.973 (0.957, 0.990) | 22513 | 4591 | 160355.83 | 28.6301 | 1.128 (1.089, 1.169) |
| **Vascular dementia** | | | | | | | | | | | |
| Q1 | | 137100 | 3779 | 942039.93 | 4.0115 | 1(Ref.) | 61107 | 1943 | 434264.41 | 4.4742 | 1(Ref.) |
| Q2 | | 154304 | 4263 | 1093020.62 | 3.9002 | 0.976 (0.934, 1.020) | 27989 | 852 | 200967.29 | 4.2395 | 0.978 (0.902, 1.060) |
| Q3 | | 156419 | 4362 | 1120847.15 | 3.8917 | 0.977 (0.935, 1.021) | 18333 | 582 | 131454.82 | 4.4274 | 1.031 (0.939, 1.132) |
| Q4 | | 163386 | 4639 | 1172810.47 | 3.9555 | 0.985 (0.942, 1.029) | 22513 | 721 | 160355.83 | 4.4963 | 1.060 (0.972, 1.156) |

^*^LDL-C quartile ranges: Q1 (LDL-C <94 mg/dl), Q2 (94 mg/dl ≤LDL-C <116 mg/dl), Q3 (116 mg/dl ≤LDL-C <139 mg/dl), Q4 (LDL-C ≥139 mg/dl).

Adjusted for age, sex, body mass index, diabetes, hypertension, current smoking status, alcohol consumption status, regular exercise, and estimated glomerular filtration rate.

LDL-C, low-density lipoprotein cholesterol; HR, hazard ratio; CI, confidence interval.

**Supplementary Table S6.** Hazard ratios stratified according to statin use for the incidence of all-cause dementia, Alzheimer’s disease, and vascular dementia according to low-density lipoprotein cholesterol level quartile among subjects aged 40–60 years.

| **LDL-C quartile^*^** | **Statin non-users** | | | | | | **Statin users** | | | | |
| --- | --- | --- | --- | --- | --- | --- | --- | --- | --- | --- | --- |
|  | **n** | | **Events (n)** | **Follow-up duration (person-years)** | **Incidence rate (per 1000 person-years)** | **HRs (95% CIs)** | **n** | **Events (n)** | **Follow-up duration (person-years)** | **Incidence rate (per 1000 person-years)** | **HRs (95% CIs)** |
| **All-cause dementia** | | | | | | | | | | | |
| Q1 | | 1084786 | 5501 | 8958766.88 | 0.61404 | 1(Ref.) | 150011 | 1781 | 1234703.79 | 1.44245 | 1(Ref.) |
| Q2 | | 1227509 | 5593 | 10177620.84 | 0.54954 | 0.875 (0.843,0.909) | 76059 | 884 | 628468.87 | 1.40659 | 0.998 (0.920,1.082) |
| Q3 | | 1198942 | 5644 | 9950793.38 | 0.56719 | 0.830 (0.799,0.862) | 58924 | 657 | 487478.38 | 1.34775 | 1.019 (0.931,1.116) |
| Q4 | | 1128175 | 6530 | 9360277.52 | 0.69763 | 0.893 (0.861,0.927) | 91550 | 1018 | 759373.88 | 1.34058 | 1.084 (1.001,1.175) |
| **Alzheimer’s disease** | | | | | | | | | | | |
| Q1 | | 1084786 | 3753 | 8958766.88 | 0.41892 | 1(Ref.) | 150011 | 1261 | 1234703.79 | 1.02130 | 1(Ref.) |
| Q2 | | 1227509 | 3881 | 10177620.84 | 0.38133 | 0.870 (0.831,0.910) | 76059 | 620 | 628468.87 | 0.98652 | 0.977 (0.887,1.076) |
| Q3 | | 1198942 | 3920 | 9950793.38 | 0.39394 | 0.814 (0.778,0.852) | 58924 | 452 | 487478.38 | 0.92722 | 0.977 (0.876,1.089) |
| Q4 | | 1128175 | 4615 | 9360277.52 | 0.49304 | 0.876 (0.838,0.916) | 91550 | 726 | 759373.88 | 0.95605 | 1.070 (0.973,1.177) |
| **Vascular dementia** | | | | | | | | | | | |
| Q1 | | 1084786 | 1289 | 8958766.88 | 0.14388 | 1(Ref.) | 150011 | 441 | 1234703.79 | 0.35717 | 1(Ref.) |
| Q2 | | 1227509 | 1366 | 10177620.84 | 0.13422 | 0.961 (0.890,1.037) | 76059 | 204 | 628468.87 | 0.32460 | 0.967 (0.818,1.142) |
| Q3 | | 1198942 | 1316 | 9950793.38 | 0.13225 | 0.894 (0.827,0.966) | 58924 | 154 | 487478.38 | 0.31591 | 1.017 (0.845,1.224) |
| Q4 | | 1128175 | 1532 | 9360277.52 | 0.16367 | 1.008 (0.934,1.087) | 91550 | 227 | 759373.88 | 0.29893 | 1.062 (0.900,1.254) |

^*^LDL-C quartile ranges: Q1 (LDL-C <94 mg/dl), Q2 (94 mg/dl ≤LDL-C <116 mg/dl), Q3 (116 mg/dl ≤LDL-C <139 mg/dl), Q4 (LDL-C ≥139 mg/dl).

Adjusted for age, sex, body mass index, diabetes, hypertension, current smoking status, alcohol consumption status, regular exercise, and estimated glomerular filtration rate.

LDL-C, low-density lipoprotein cholesterol; HR, hazard ratio; CI, confidence interval.

**Supplementary Table S7.** Hazard ratios stratified according to statin use for the incidence of all-cause dementia, Alzheimer’s disease, and vascular dementia according to low-density lipoprotein cholesterol level quartile among subjects aged >60 years.

| **LDL-C quartile^*^** | **Statin non-users** | | | | | | **Statin users** | | | | |
| --- | --- | --- | --- | --- | --- | --- | --- | --- | --- | --- | --- |
|  | **n** | | **Events (n)** | **Follow-up duration (person-years)** | **Incidence rate (per 1000 person-years)** | **HRs (95% CIs)** | **n** | **Events (n)** | **Follow-up duration (person-years)** | **Incidence rate (per 1000 person-years)** | **HRs (95% CIs)** |
| **All-cause dementia** | | | | | | | | | | | |
| Q1 | | 331139 | 42555 | 2473836.8 | 17.20200 | 1(Ref.) | 150454 | 20336 | 1145069.35 | 17.7596 | 1(Ref.) |
| Q2 | | 380340 | 46876 | 2907568.92 | 16.12210 | 0.936(0.923,0.948) | 71056 | 9596 | 545992.69 | 17.5753 | 1.014(0.99,1.039) |
| Q3 | | 399505 | 47918 | 3083828.87 | 15.53850 | 0.917(0.905,0.929) | 47936 | 6606 | 368465.73 | 17.9284 | 1.056(1.027,1.086) |
| Q4 | | 426533 | 53038 | 3301805.25 | 16.06330 | 0.944(0.932,0.956) | 60575 | 8652 | 465775.58 | 18.5755 | 1.114(1.086,1.143) |
| **Alzheimer’s disease** | | | | | | | | | | | |
| Q1 | | 331139 | 34234 | 2473836.8 | 13.83840 | 1(Ref.) | 150454 | 16208 | 1145069.35 | 14.1546 | 1(Ref.) |
| Q2 | | 380340 | 37873 | 2907568.92 | 13.02570 | 0.935(0.921,0.948) | 71056 | 7749 | 545992.69 | 14.1925 | 1.021(0.994,1.049) |
| Q3 | | 399505 | 38877 | 3083828.87 | 12.60670 | 0.918(0.905,0.932) | 47936 | 5261 | 368465.73 | 14.2781 | 1.047(1.015,1.081) |
| Q4 | | 426533 | 43127 | 3301805.25 | 13.06160 | 0.945(0.931,0.958) | 60575 | 6984 | 465775.58 | 14.9943 | 1.116(1.084,1.148) |
| **Vascular dementia** | | | | | | | | | | | |
| Q1 | | 331139 | 5794 | 2473836.8 | 2.34211 | 1(Ref.) | 150454 | 3097 | 1145069.35 | 2.70464 | 1(Ref.) |
| Q2 | | 380340 | 6407 | 2907568.92 | 2.20356 | 0.96(0.927,0.995) | 71056 | 1382 | 545992.69 | 2.53117 | 0.984(0.923,1.049) |
| Q3 | | 399505 | 6525 | 3083828.87 | 2.11588 | 0.944(0.911,0.979) | 47936 | 961 | 368465.73 | 2.60811 | 1.042(0.969,1.121) |
| Q4 | | 426533 | 7011 | 3301805.25 | 2.12338 | 0.955(0.922,0.99) | 60575 | 1176 | 465775.58 | 2.52482 | 1.044(0.975,1.118) |

^*^LDL-C quartile ranges: Q1 (LDL-C <94 mg/dl), Q2 (94 mg/dl ≤LDL-C <116 mg/dl), Q3 (116 mg/dl ≤LDL-C <139 mg/dl), Q4 (LDL-C ≥139 mg/dl).

Adjusted for age, sex, body mass index, diabetes, hypertension, current smoking status, alcohol consumption status, regular exercise, and estimated glomerular filtration rate.

LDL-C, low-density lipoprotein cholesterol; HR, hazard ratio; CI, confidence interval.

**Supplementary Table S8.** Hazard ratios stratified according to diabetes status among never-users, irregular or short-term users, and continuous users of statins for the incidence of all-cause dementia according to quartiles of low-density lipoprotein cholesterol level.

| **LDL-C quartile^*^** | **Individuals without diabetes** | | | | | | **Individuals with diabetes** | | | | |
| --- | --- | --- | --- | --- | --- | --- | --- | --- | --- | --- | --- |
|  | **n** | | **Events (n)** | **Follow-up duration (person-years)** | **Incidence rate (per 1000 person-years)** | **HRs (95% CIs)** | **n** | **Events (n)** | **Follow-up duration (person-years)** | **Incidence rate (per 1000 person-years)** | **HRs (95% CIs)** |
| **Among never-users of statin** | | | | | | | | | | | |
| Q1 | | 1207110 | 33758 | 9809259.27 | 3.44144 | 1(Ref.) | 130834 | 9023 | 1009971.32 | 8.9339 | 1(Ref.) |
| Q2 | | 1397012 | 37742 | 11424779.00 | 3.30352 | 0.926 (0.912,0.939) | 120422 | 8357 | 943406.90 | 8.8583 | 0.934 (0.906,0.962) |
| Q3 | | 1359503 | 37532 | 11137155.23 | 3.36998 | 0.907 (0.893,0.920) | 112513 | 7647 | 887696.80 | 8.6144 | 0.921 (0.893,0.950) |
| Q4 | | 1215387 | 37610 | 9950145.89 | 3.77984 | 0.936 (0.922,0.950) | 105273 | 7090 | 832763.99 | 8.5138 | 0.929 (0.900,0.959) |
| **Among irregular or short-term users of statin** | | | | | | | | | | | |
| Q1 | | 74352 | 4122 | 595400.45 | 6.92307 | 1(Ref.) | 30257 | 2499 | 234716.38 | 10.6469 | 1(Ref.) |
| Q2 | | 75742 | 4423 | 608788.30 | 7.26525 | 0.990 (0.949,1.033) | 22031 | 1990 | 171114.49 | 11.6296 | 0.996 (0.939,1.056) |
| Q3 | | 103831 | 5632 | 839236.96 | 6.71086 | 0.936 (0.899,0.974) | 24430 | 2283 | 190252.92 | 11.9998 | 1.003 (0.947,1.062) |
| Q4 | | 206571 | 10621 | 1676333.05 | 6.33585 | 0.934 (0.901,0.969) | 34998 | 3277 | 273457.32 | 11.9836 | 0.992 (0.941,1.046) |
| **Among continuous-users of statin** | | | | | | | | | | | |
| Q1 | | 159185 | 10684 | 1268341.46 | 8.42360 | 1(Ref.) | 114652 | 10087 | 894687.95 | 11.2743 | 1(Ref.) |
| Q2 | | 92712 | 6217 | 743208.71 | 8.36508 | 1.007 (0.976,1.039) | 47045 | 4220 | 368353.93 | 11.4564 | 1.003 (0.967,1.039) |
| Q3 | | 72489 | 4792 | 581821.78 | 8.23620 | 1.023 (0.989,1.059) | 32541 | 2939 | 254402.67 | 11.5526 | 1.022 (0.980,1.064) |
| Q4 | | 107729 | 6903 | 867996.98 | 7.95279 | 1.055 (1.023,1.088) | 36875 | 3737 | 286535.02 | 13.0420 | 1.100 (1.059,1.142) |

^*^LDL-C quartile ranges: Q1 (LDL-C <94 mg/dl), Q2 (94 mg/dl ≤LDL-C <116 mg/dl), Q3 (116 mg/dl ≤LDL-C <139 mg/dl), Q4 (LDL-C ≥139 mg/dl).

Adjusted for age, sex, body mass index, diabetes, hypertension, current smoking status, alcohol consumption status, regular exercise, and estimated glomerular filtration rate.

LDL-C, low-density lipoprotein cholesterol; HR, hazard ratio; CI, confidence interval.

**Supplementary Figure S1.** Hazard ratios for incidence of all-cause dementia, Alzheimer’s disease, and vascular dementia among statin users with atherosclerotic cardiovascular disease according to decile of low-density lipoprotein cholesterol level.





Adjusted for age, sex, body mass index, hypertension, current smoking status, alcohol consumption status, regular exercise, and estimated glomerular filtration rate.

*LDL-C decile ranges: D1 (LDL-C <75 mg/dl), D2 (75 mg/dl ≤LDL-C <89 mg/dl), D3 (89 mg/dl ≤LDL-C <99 mg/dl), D4 (99 mg/dl ≤LDL-C <107 mg/dl), D5 (107 mg/dl ≤LDL-C <116 mg/dl), D6 (116 mg/dl ≤LDL-C <124 mg/dl), D7 (124 mg/dl ≤LDL-C <133 mg/dl), D8 (133 mg/dl ≤LDL-C <145 mg/dl), D9 (145 mg/dl ≤LDL-C <162 mg/dl), D10 (LDL-C ≥162 mg/dl).

LDL-C, low-density lipoprotein cholesterol; CI, confidence interval.
